# Supplementary material for: Structural basis for DARC binding in reticulocyte invasion by Plasmodium vivax
Source: Nat Commun. 2023 Jun 19;14:3637. doi: 10.1038/s41467-023-39357-w (PMC10279640; doi:10.1038/s41467-023-39357-w)
Supplement: Supplementary file 1 — Supplementary information [file 41467_2023_39357_MOESM1_ESM.pdf]

Supplementary Information

Structural basis for DARC binding in reticulocyte invasion by *Plasmodium vivax*

Re'em Moskovitz[1,†], Tossapol Pholcharee[1,†], Sophia M. DonVito[2], Bora Guloglu[1], Edward Lowe[1], Franziska Mohring[2], Robert W. Moon[2] and Matthew K. Higgins[1,*]

[1] Department of Biochemistry, University of Oxford, South Parks Road, Oxford, OX1 3QU, UK

[2] London School of Hygiene and Tropical Medicine, Keppel Street, London, WC1E 7HT, UK

† These authors contributed equally

* Correspondence should be addressed to: matthew.higgins@bioch.ox.ac.uk

Supplementary Table 1: Crystallographic statistics

**Data collection**	
Space group	$P2_12_12_1$
Cell dimensions:	
a, b, c (Å)	52.20, 97.95, 287.99
α, β, γ (°)	90, 90, 90
Resolution (Å)	92.73 – 2.49 (2.57 – 2.49)
Total observations	99952 (8634)
Total unique	52982 (4488)
R_{pim}	0.070 (0.735)
$CC_{1/2}$	0.993 (0.463)
I/σ(I)	5.7 (0.7)
Completeness (%)	100 (100)
Multiplicity	1.9 (1.9)
Wilson B factor ($Å^2$)	56.4
**Refinement**	
Number of reflections	52884
R_{work} / R_{free}	0.198 / 0.225
Average B factor ($Å^2$)	67
Chain A	72
Chain B	85
Chain D	52
Chain E	58
Number of residues:	
Amino acid residues	760
Waters	250
RMSZ deviations	
Bond lengths	0.008
Bond angles	0.985
Ramachandran plot	
Favoured (%)	96.3
Allowed (%)	3.7
Outliers (%)	0

Supplementary Table 2: interactions

PvDBP-RII			DARC			
**Chain**	**Residue**	**Group**	**Chain**	**Residue**	**Group**	**Type of interaction**
B	L20	Side chain	A	V282/Y363	Side chain	Hydrophobic
B	F22	Side chain	A	Y278/V282	Side chain	Hydrophobic
B	D24	Side chain	A	Y363	Side chain	Hydrogen bond
B	D24	Side chain	A	K367	Side chain	Electrostatic
B	V25	Side chain	A	A281/Y363	Side chain	Hydrophobic
B	W26	Side chain	A	R274	Side chain	Cation- π
B	S29	Side chain	A	Q356	Side chain	Hydrogen bond
B	D34	Side chain	A	K366	Side chain	Electrostatic
B	F36	Side chain	A	F373	Side chain	Hydrophobic
B	P37	Main chain	A	W375	Main chain	Hydrogen bond
B	G39	Main chain	A	W375	Main chain	Hydrogen bond
B	Y41S	Side chain	A	K297/I376	Side chain	Hydrophobic
B	Y41S	Side chain	A	K301/R304	Side chain	Electrostatic
B	N44	Side chain	A	R294/N296	Side chain	Hydrogen bond
B	L45	Main chain	A	Y295	Main chain	Hydrogen bond
B	A47	Main chain	A	Y293	Main chain	Hydrogen bond

PvDBP-RII			DB1			
**Chain**	**Residue**	**Group**	**Chain**	**Residue**	**Group**	**Type of interaction**
A	N260	Side chain	D	S75	Side chain	Hydrogen bond
A	F261	Main chain	D	N74	Side chain	Hydrogen bond
A	F261	Side chain	D	Y68	Side chain	Hydrophobic
A	H262	Side chain	C	Y120	Main chain	Hydrogen bond
A	H262	Side chain	C	Y51	Side chain	Hydrogen bond
A	R263	Side chain	C	S122	Side chain	Hydrogen bond
A	R333	Side chain	C	Y125	Side chain	Hydrogen bond
A	I335	Main chain	C	Y120	Side chain	Hydrogen bond
A	T338	Main chain	C	Y127	Side chain	Hydrogen bond
A	D339	Main chain	C	Y127	Side chain	Hydrogen bond
A	D339	Side chain	C	Y127	Side chain	Hydrogen bond
A	D339	Side chain	C	R345	Side chain	Hydrogen bond
A	D339	Side chain	C	W52	Side chain	Hydrogen bond
A	E340	Side chain	C	R78	Side chain	Electrostatic
A	K341	Side chain	C	D74	Side chain	Electrostatic
A	K341	Side chain	C	D76	Side chain	Electrostatic
A	R345	Side chain	C	Y71	Side chain	Hydrogen bond

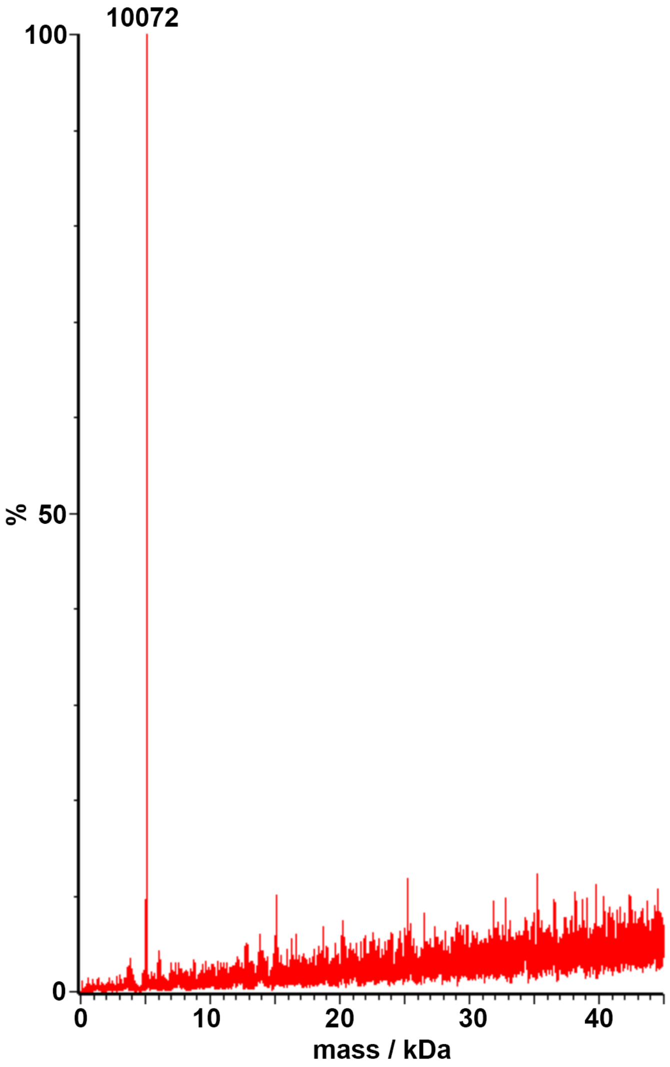

Supplementary Figure 1: characterisation of DARC peptide

The DARC peptide expressed in HEK293 cells (TMGNALHRAELSPSTENSSQLDFEDVWNSSY GVNDSFPDGDYDANLEAAAPAHSANLLDDSLVPRGSGLNDIFEAQKIEWHEGSHHHHHH) was assessed by mass spectrometry. The mass of the fully-protonated peptide would be 9918 and the mass of 10072 was consistent with a peptide with two modifications by sulphation.

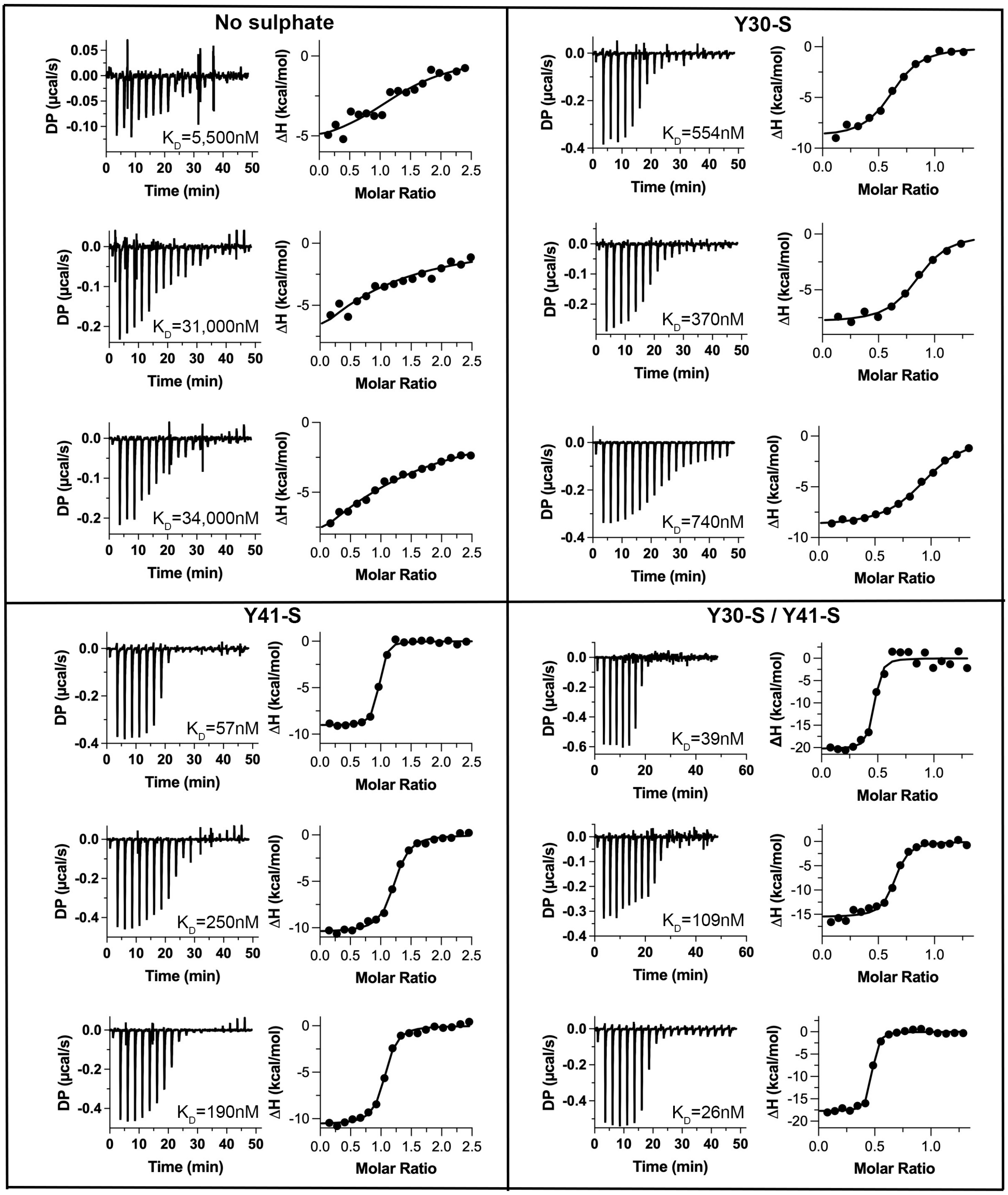

Supplementary Figure 2: Measurement of affinities of PvDBP-RII for DARC peptides by isothermal titration calorimetry

In each case, three separate technical replicates were performed and the mean and standard deviation K_D value was determined. For the non-sulphated peptide, the measured K_D values were 5.5 ± 6.4 µM, 31 ± 28 µM and 34 ± 40 µM with the mean of 23 ± 16 µM. For the Y30-S peptide, the measured K_D values were 544 ± 116 nM, 370 ± 120 nM and 740 ± 84 nM with the mean of 552 ± 186 nM. For the Y41-S peptide, the measured K_D values were 57 ± 13 nM, 250 ± 45 nM and 190 ± 34 nM with the mean of 168 ± 101 nM. For the Y30-S / Y41-S peptide, the measured K_D values were 39 ± 25 nM, 109 ± 33 nM and 26 ± 7 nM with the mean of 58 ± 45 nM. Source data are provided as a Source Data file.

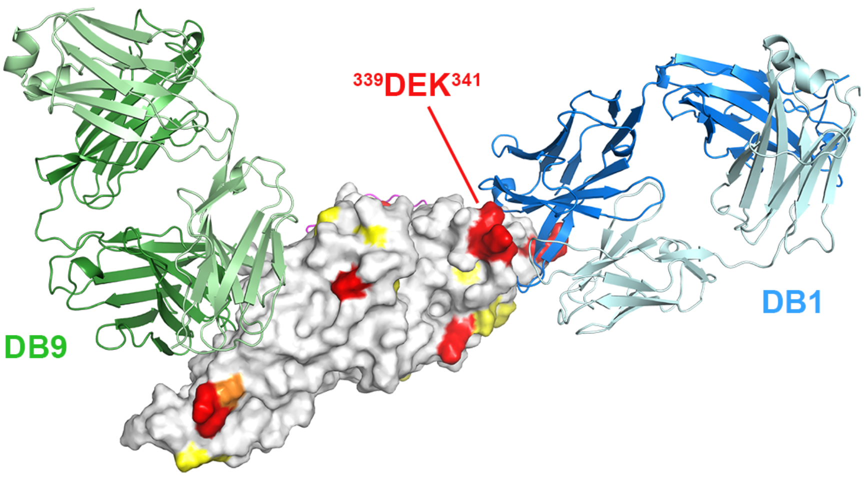

Supplementary Figure 3: Polymorphism in the epitopes for DB1 and DB9
The structure of PvDBP-RII is shown in surface representation, which has been coloured according to sequence polymorphism (grey <0.15; yellow = 0.15-0.30; orange = 0.30-0.40 and red > 0.4). The Fab fragments of DB9 (green) and DB1 (blue) are shown. DB9 binds to a non-polymorphic site, while DB1 binds directly to the polymorphic ^{339}DEK^{341} motif.

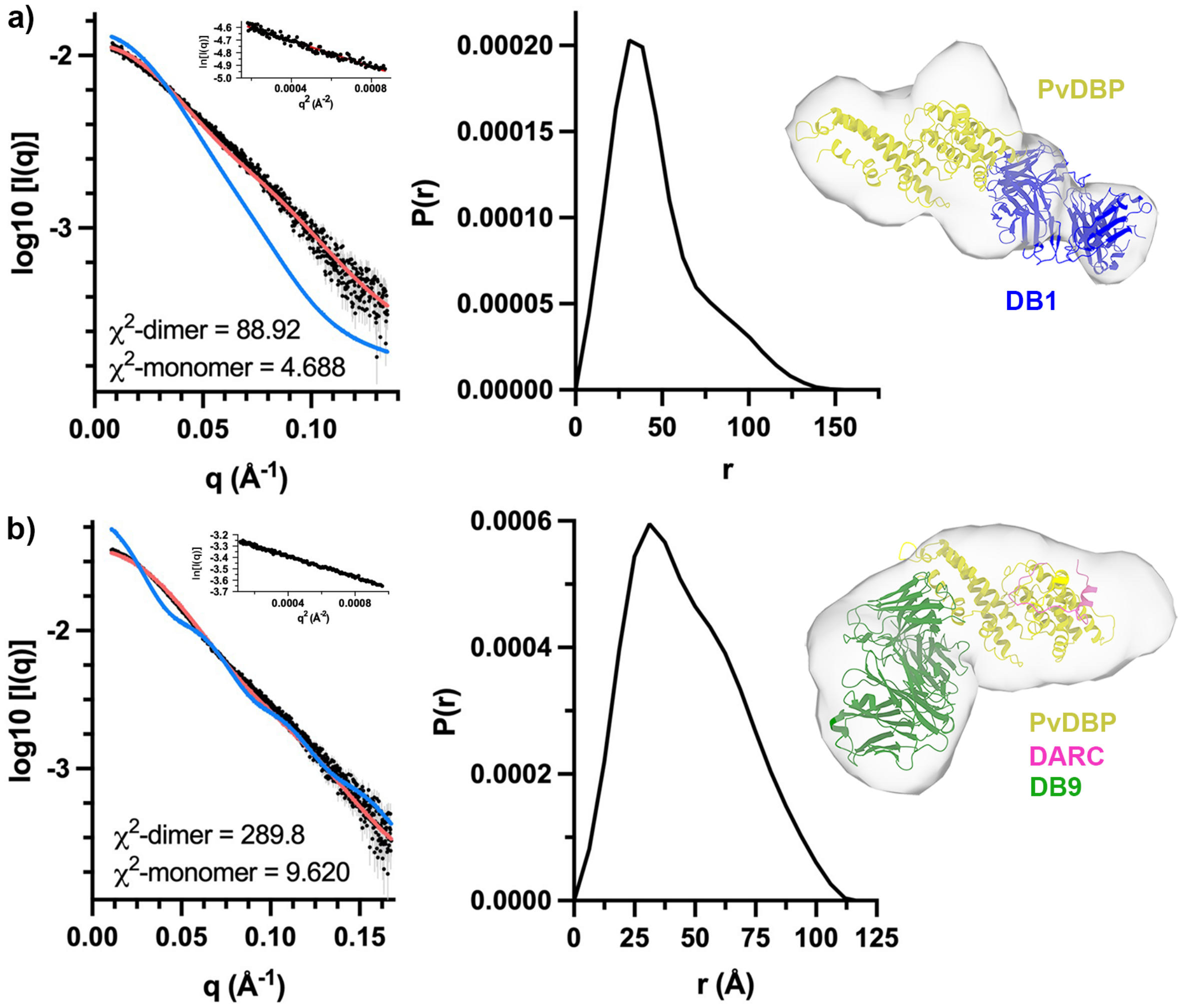

Supplementary Figure 4: Small angle X-ray scattering data (SEC-SAXS) for PvDBP-RII complexes

Small angle X-ray scattering data (SEC-SAXS) for **a)** the complex of PvDBP-RII and the Fab fragment of antibody DB1 and **b)** the complex of PvDBP-RII, Y30-S, Y41-S double-sulphated $DARC_{19-47}$ and the Fab fragment of antibody DB9. In each case a single sample was studied and the left-hand panel shows the refined scattering data, with black circles representing the mean and error bars representing the standard error after radial averaging of intensities from two-dimensional diffraction data. This has been fitted either with the predicted scattering for the monomeric (red) or dimeric (blue) complex. The inset is the Guinier plot. The central panel shows the pair distance distribution function while the right-hand panel shows the calculated envelope into which has been docked the monomeric model. Source data are provided as a Source Data file.

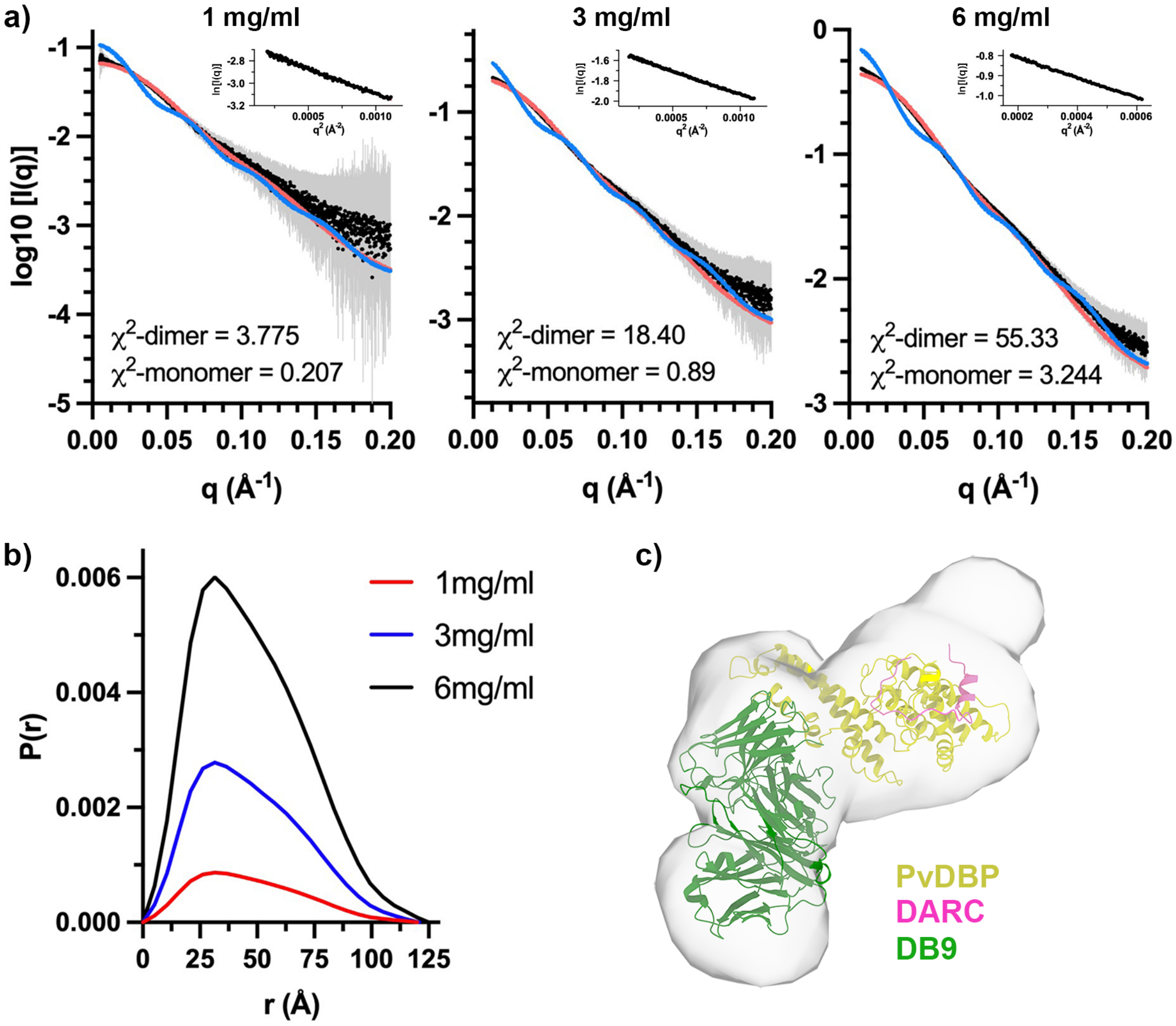

Supplementary Figure 5: Small angle X-ray scattering data for PvDBP-RII:DARC$_{19\text{-}47}$:DB9 complexes in batch model

a) Small angle X-ray scattering data (SEC-SAXS) for the complex of PvDBP-RII, DARC$_{19\text{-}47}$ and the Fab fragment of antibody DB9 collected in batch mode at 1mg/ml (left), 3mg/ml (central) and 6mg/ml (right). In each case, a single sample was studied and the refined scattering data is shown with black circles representing the mean and error bars representing the standard error after radial averaging of intensities from two-dimensional diffraction data. This has been fitted either with the predicted scattering for the monomeric (red) or dimeric (blue) complex. The insets are the representative Guinier plots. **b)** The pair distance distribution functions for the 1mg/ml (red), 3mg/ml (blue) and 6mg/ml (black) data. **c)** The calculated envelope into which has been docked the monomeric model from the 3mg/ml data. Source data are provided as a Source Data file.
