# Supplementary material for: Structural basis for DARC binding in reticulocyte invasion by Plasmodium vivax
Source: Nat Commun. 2023 Jun 19;14:3637. doi: 10.1038/s41467-023-39357-w (PMC10279640; doi:10.1038/s41467-023-39357-w)
Supplement: Supplementary file 3 — Reporting Summary [file 41467_2023_39357_MOESM3_ESM.pdf]

nature research

Corresponding author(s): Matthew K. Higgins

Last updated by author(s): 28 May 2023

Reporting Summary

Nature Research wishes to improve the reproducibility of the work that we publish. This form provides structure for consistency and transparency in reporting. For further information on Nature Research policies, see our Editorial Policies and the Editorial Policy Checklist.

Statistics

For all statistical analyses, confirm that the following items are present in the figure legend, table legend, main text, or Methods section.

n/a	Confirmed	
☐	☒	The exact sample size (*n*) for each experimental group/condition, given as a discrete number and unit of measurement
☐	☒	A statement on whether measurements were taken from distinct samples or whether the same sample was measured repeatedly
☒	☐	The statistical test(s) used AND whether they are one- or two-sided<br>*Only common tests should be described solely by name; describe more complex techniques in the Methods section.*
☒	☐	A description of all covariates tested
☒	☐	A description of any assumptions or corrections, such as tests of normality and adjustment for multiple comparisons
☐	☒	A full description of the statistical parameters including central tendency (e.g. means) or other basic estimates (e.g. regression coefficient) AND variation (e.g. standard deviation) or associated estimates of uncertainty (e.g. confidence intervals)
☒	☐	For null hypothesis testing, the test statistic (e.g. *F*, *t*, *r*) with confidence intervals, effect sizes, degrees of freedom and *P* value noted<br>*Give P values as exact values whenever suitable.*
☒	☐	For Bayesian analysis, information on the choice of priors and Markov chain Monte Carlo settings
☒	☐	For hierarchical and complex designs, identification of the appropriate level for tests and full reporting of outcomes
☒	☐	Estimates of effect sizes (e.g. Cohen's *d*, Pearson's *r*), indicating how they were calculated

Our web collection on statistics for biologists contains articles on many of the points above.

Software and code

Policy information about availability of computer code

Data collection	For crystal structure determination, the structure was solved by molecular replacement with Phaser MR (v.2.8.3) using a search model for PvDBP-RII (PDB: 6R2S). The model was built and refined using cycles of COOT (v.0.8.9.2) and BUSTER (v.2.10).
Data analysis	Data analysis for ITC data in Figure 3 was analyzed using the MicroCal PEAQ-ITC Analysis Software v1.3 (Malvern) provided with the instrument, then all data were exported to and plotted in GraphPad Prism 9. For the growth-inhibition assay data in the same figure, IC50 values were identified using a variable slope four-parameter logistic curve, calculated using GraphPad Prism 9. The SAXS data in supplementary figure 3-4 were processed using ScÅtter with the ATSAS software suites. v3.1.0-1

For manuscripts utilizing custom algorithms or software that are central to the research but not yet described in published literature, software must be made available to editors and reviewers. We strongly encourage code deposition in a community repository (e.g. GitHub). See the Nature Research guidelines for submitting code & software for further information.

Data

Policy information about availability of data

All manuscripts must include a data availability statement. This statement should provide the following information, where applicable:

- Accession codes, unique identifiers, or web links for publicly available datasets
- A list of figures that have associated raw data
- A description of any restrictions on data availability

Coordinates and structure factors are deposited in the Protein Data Bank with accession code 8A44 and all other raw data is included in a Source Data File which accompanies the manuscript.

Field-specific reporting

Please select the one below that is the best fit for your research. If you are not sure, read the appropriate sections before making your selection.

☒ Life sciences ☐ Behavioural & social sciences ☐ Ecological, evolutionary & environmental sciences

For a reference copy of the document with all sections, see nature.com/documents/nr-reporting-summary-flat.pdf

Life sciences study design

All studies must disclose on these points even when the disclosure is negative.

Sample size	The majority of the data present in this manuscript is structural and biophysical data for well-defined purified protein samples and sample sizes were determined based on past experience. For parasite growth-inhibition experiments, past experience indicated that four biological replicates with two technical replicates would provide statistically significant outcomes.
Data exclusions	No data were excluded from the analysis
Replication	Isothermal titration calorimetry was performed in triplicate, all of which display consistent results. For parasite growth-inhibition experiments, technical replicates (n=2) from each assay were averaged, and data presented represents the mean ± standard error of the mean of four separate biological replicates. All attempts at replication were successful. Molecular dynamics simulations were conducted in three independent simulations. Structural biology data was each collected once.
Randomization	Randomisation was not conducted as all experiments resulted in quantitative outputs and were not subjective.
Blinding	Blinding was not conducted, as all experiments had a quantitative outcome and were not subject user-based bias.

Reporting for specific materials, systems and methods

We require information from authors about some types of materials, experimental systems and methods used in many studies. Here, indicate whether each material, system or method listed is relevant to your study. If you are not sure if a list item applies to your research, read the appropriate section before selecting a response.

Materials & experimental systems

n/a	Involved in the study	
☐	☒	Antibodies
☐	☒	Eukaryotic cell lines
☒	☐	Palaeontology and archaeology
☒	☐	Animals and other organisms
☒	☐	Human research participants
☒	☐	Clinical data
☒	☐	Dual use research of concern

Methods

n/a	Involved in the study	
☒	☐	ChIP-seq
☒	☐	Flow cytometry
☒	☐	MRI-based neuroimaging

Antibodies

Antibodies used	Antibodies DB1 and DB9 are described in Rawlinson et al, 2019, as cited. They were originally cloned from human volunteers vaccinated with a PvDBP-RII vaccine. The antibodies used in this study were expressed, purified and characterised by the authors and are used at the concentrations indicated in the manuscript.
Validation	Antibodies DB1 and DB9 were validated in Rawlinson et al, 2019.

Eukaryotic cell lines

Policy information about cell lines

Cell line source(s)	Human embryonic kidney (HEK293_F and Expi HEK293-F) cells are from ThermoFisher Scientific.
Authentication	The cells were not authenticated before.
Mycoplasma contamination	The cell line used was not tested for mycoplasma contamination as the cells were used for protein expression rather than to obtain experimental outcomes.
Commonly misidentified lines (See ICLAC register)	No commonly misidentified cell lines were used in the study.
